# Increase in Sexually Transmitted Infections among Men Who Have Sex with Men, England, 2014

**DOI:** 10.3201/eid2201.151331

**Published:** 2016-01

**Authors:** Hamish Mohammed, Holly Mitchell, Bersabeh Sile, Stephen Duffell, Anthony Nardone, Gwenda Hughes

**Affiliations:** Public Health England, London, UK

**Keywords:** syphilis, gonorrhea, men who have sex with men, surveillance, HIV/AIDS and other retroviruses, bacteria, sexually transmitted infections, viruses, England

## Abstract

Surveillance data from sexual health clinics indicate recent increases in sexually transmitted infections, particularly among men who have sex with men. The largest annual increase in syphilis diagnoses in a decade was reported in 2014. Less condom use may be the primary reason for these increases.

Sexually transmitted infections (STIs) are a major public health concern; they can facilitate the transmission of HIV and are associated with severe disease. Treatment for some STIs, especially gonorrhea, has been compromised by antimicrobial drug resistance (*1*). In 2014 in England, there were 439,243 diagnoses of STIs. Although this number reflects a very small decline (0.3%) relative to 2013, numbers of diagnoses of syphilis and gonorrhea rose substantially, by 33% (from 3,236 to 4,317) and 19% (from 29,419 to 34,958), respectively (*2*). This number of syphilis diagnoses is the highest reported in England since 1949, and the number of gonorrhea diagnoses is the highest reported since 1986. These increases resulted almost entirely from increased diagnoses among men who have sex with men (MSM), among whom diagnoses of syphilis and gonorrhea increased 46% (from 2,375 to 3,477) and 32% (from 13,629 to 18,029), respectively (Figure 1), resulting in the highest number of diagnoses of these STIs since reporting among MSM began in 1994. We explored the epidemiology of these and other STIs among MSM and describe recent trends.

**Figure 1:**
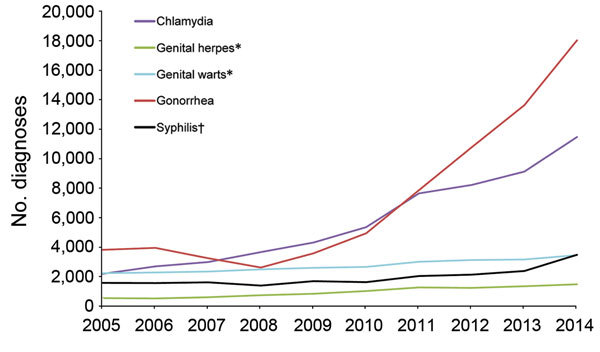
New diagnoses of selected sexually transmitted infections among men who have sex with men who attended sexual health clinics, England, 2005–2014. *First episode. †Primary, secondary, or early latent.

## The Study

In England, surveillance for STIs is conducted through mandatory reporting in sexual health clinics (SHCs) (genitourinary medicine [GUM] and integrated GUM/sexual and reproductive health clinics) by using the GUM clinic activity dataset version 2 (GUMCADv2) (*3*). SHCs provide free and open access services and, since 2012, all (216 in 2014) have submitted data to Public Health England. Although STIs are not notifiable diseases in the United Kingdom, GUMCADv2 is a comprehensive, patient-level dataset of all attendances and laboratory-confirmed STIs at SHCs. Information about the sexual orientation of each attendee is collected through GUMCADv2 (completion >90% since 2011); this information was also collected by the preceding system, the KC60 aggregate return (*3*). We reviewed the most recent GUMCADv2 data, extracted on April 28, 2015, to assess trends in laboratory-confirmed gonorrhea, infectious (primary/secondary/early latent) syphilis, chlamydia, genital herpes (first episode), and genital warts (first episode). Only 1 diagnosis of each STI was counted within a 6-week period; this restriction also applies to instances of infection at multiple anatomic sites (*3*). These data represent the number of diagnoses reported, not the number of persons in whom the infections were diagnosed. Descriptive analyses by demographic characteristics and tests for linear trend were performed; p values <5% were considered significant.

Since 2013, syphilis diagnoses among MSM increased by 46% (from 2,375 to 3,477); this increase is the largest year-on-year increase in syphilis diagnoses among MSM since 2005 (Figure 1; Table 1). Relative to 2013, in 2014 the number of syphilis diagnoses among heterosexual men and men of unknown sexual orientation decreased by 0.2% (from 578 to 577), with a larger decrease in women (7.1%, from 283 to 263) (Table 1).

**Table 1:** Number of diagnoses of selected STIs made at sexual health clinics, England*

STI	Patient category	2005	2006	2007	2008	2009	2010	2011	2012	2013	2014	% Increase, 2014 vs. 2013
Syphilis	MSM	1,569	1,560	1,610	1,387	1,692	1,618	2,036	2,129	2,375	3,477	46.4
	Non-MSM men	1,114	1,123	1,173	1,121	808	733	598	569	578	577	−0.2
	Women	503	433	424	366	345	292	291	261	283	263	−7.1
Gonorrhea	MSM	3,817	3,945	3,245	2,615	3,579	4,938	7,860	10,768	13,629	18,029	32.3
	Non-MSM men	8,707	8,142	8,340	7,205	7,250	6,696	7,221	7,815	8,122	8,546	5.2
	Women	5,108	5,104	5,534	5,165	5,299	5,198	6,007	6,992	7,664	8,379	9.3
Chlamydia	MSM	2,183	2,693	2,982	3,658	4,313	5,349	7,644	8,215	9,118	11,468	25.8
	Non-MSM men	43,122	46,363	50,584	50,892	46,169	42,669	44,405	43,327	44,512	44,339	−0.4
	Women	51,986	51,321	54,941	55,872	50,125	46,080	50,048	45,870	48,642	51,045	4.9
Genital herpes	MSM	538	515	600	735	834	1,019	1,264	1,233	1,339	1,474	10.1
	Non-MSM men	6,192	6,941	8,455	9,369	9,985	10,563	10,662	10,861	10,938	10,415	−4.8
	Women	10,649	11,798	14,432	15,990	16,604	18,101	19,226	19,770	20,069	19,883	−0.9
Genital warts	MSM	2,225	2,280	2,339	2,488	2,592	2,657	3,004	3,120	3,156	3,456	9.5
	Non-MSM men	33,750	34,741	37,384	38,606	39,308	38,238	38,596	37,272	37,872	35,893	−5.2
	Women	31,877	32,679	35,549	37,062	35,931	34,659	34,935	33,493	32,834	31,251	−4.8

*Genitourinary medicine (GUM) and integrated GUM/sexual and reproductive health clinics. In 2014 unknown sexual orientation was reported for < 4% of men. Figures for women who have sex with women are not reported here because in 2014, homosexuality was reported for <0.5% of women. MSM, men who have sex with men; non-MSM men, heterosexual men and men of unknown sexual orientation; STI, sexually transmitted infection.

From 2013 to 2014, diagnoses of gonorrhea among MSM increased 32% (from 13,629 to 18,029) (Figure 1; Table 1). Although this increase was relatively large, it is consistent with the increasing trend from 2005 (3,817 diagnoses; p = 0.015). Compared with the increased diagnoses in MSM since 2013, in 2014 the increases in gonorrhea diagnoses among heterosexual men and men of unknown sexual orientation (5.2%, from 8,122 to 8,546) and women (9.3%, from 7,664 to 8,379) were smaller (Table 1).

Relative to 2013, in 2014, diagnoses further increased for chlamydia (25.8%), genital herpes (10.1%), and genital warts (9.5%) among MSM, consistent with the increasing trend since 2005 (Figure 1; Table 1). Diagnoses of chlamydia decreased among heterosexual men and men of unknown sexual orientation (0.4%, from 44,512 to 44,339) and increased among women (4.9%, from 48,642 to 51,045). Genital herpes diagnoses decreased by 4.8% (from 10,938 to 10,415) and 0.9% (from 20,069 to 19,883) and genital warts by 5.2% (from 37,872 to 35,893) and 4.8% (from 32,834 to 31,251) among heterosexual men and men of unknown sexual orientation and among women, respectively.

**Figure 2:**
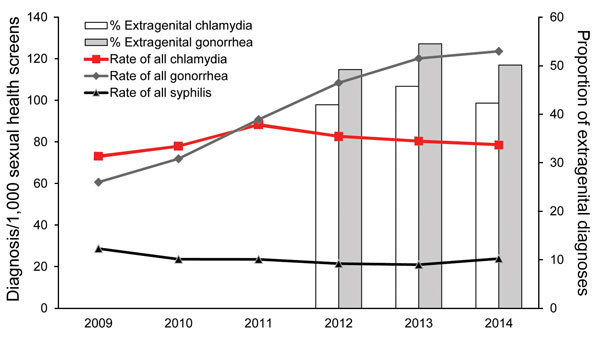
Rate of diagnoses (per 1,000 sexual health screens) of chlamydia, gonorrhea, and syphilis (primary, secondary, and early latent) and proportion of extragenital chlamydia and gonorrhea diagnoses among men who have sex with men who attended sexual health clinics, England, 2009–2014. Surveillance codes for extragenital infections were introduced mid-2011 and are only available for chlamydia and gonorrhea diagnoses.

Although the number of sexual health screen (tests for chlamydia, gonorrhea, HIV, and syphilis) among MSM increased 29% from 2013 to 2014, the rates (diagnoses/1,000 screenings) of syphilis and gonorrhea increased 13.8% (from 20.9 to 23.8) and 2.9% (from 120.2 to 123.6), respectively. The rate (diagnosis/1,000 screenings) of chlamydia decreased 2.2% (from 80.4 to 78.6) (Figure 2), and the proportion of diagnoses of extragenital chlamydia and gonorrhea also decreased (Figure 2).

In 2014, the median age for MSM in whom these STIs were diagnosed ranged from 28 years (genital warts) to 36 years (syphilis) (Table 2). More than three quarters of MSM in whom STIs were diagnosed were white, 60.3%–71.4% were born in the United Kingdom, and 12.6%–18.4% were born in other European countries (Table 2). MSM in whom bacterial STIs were diagnosed were more likely to be HIV positive and live in London than were MSM in whom genital warts and herpes were diagnosed (Table 2).

**Table 2:** Diagnoses of selected sexually transmitted infections among men who have sex with men attending sexual health clinics, by patient characteristics, England, 2014*

Patient characteristic	Syphilis†	Gonorrhea	Chlamydia	Genital herpes‡	Genital warts‡	No STI§
No. diagnoses	3,477	18,029	11,468	1,474	3,456	NA
Median age (interquartile range), y	36 (29–44)	31 (25–38)	32 (26–41)	31 (25–38)	28 (23–36)	34 (26–44)
% White or white British	78.9	78.8	77.4	80.1	82.6	80.4
% London residents	58.9	60.3	56.3	49.7	40.4	48.7
% Born in the United Kingdom	60.3	61.0	61.6	69.5	71.4	65.9
% Born in Europe outside the United Kingdom)	18.4	18.2	16.6	12.6	12.6	14.5
% HIV positive	44.3	22.9	26.1	18.4	8.0	29.6

*Genitourinary medicine (GUM) and integrated GUM/sexual and reproductive health clinics. NA, not applicable. †Primary, secondary, or early latent. ‡First episode. §N = 2,549,652 attendances at SHCs where no sexually transmitted infection was diagnosed.

## Conclusions

We report the continuing increase of diagnoses of all STIs among MSM, particularly for syphilis, for which the largest number of cases was recorded since 1994. Previously reported trends (*4*) markedly worsened in 2014. Similar levels of syphilis diagnoses among men (reporting for MSM began in 1994) were last reported in the late 1970s and were followed by a precipitous decline after the emergence of HIV in the United Kingdom in the 1980s (*5*). In 2014, most MSM in whom STIs were diagnosed lived in London, and an average of 16% were born in Europe outside the United Kingdom. Given such a mobile population, the potential for spread of these infections to MSM in other major cities is clear (*6*).

Diagnoses of STIs among HIV-positive MSM since 2009 have steadily increased; the STI rate is 2–4 times that among MSM who are HIV negative or of unknown HIV status (*7*). Sex without condom use, associated with increasing HIV seroadaptive behaviors (i.e., seeking out partners according to their HIV serostatus for unprotected sex) and use of geospatial social networking applications, may facilitate STI transmission in concentrated sexual networks (*8*,*9*). Given the risk for emergence of strains of *Neisseria gonorrhoeae* that are resistant to first-line antimicrobial drugs (*1*), the increase in gonorrhea diagnoses, especially among MSM, is concerning.

In response to gonorrhea testing guidance published in 2010 (*10*), use of highly sensitive nucleic acid amplification tests for screening of extragenital sites in MSM has occurred more frequently (*11*); this change may account for part of the increase in gonorrhea diagnoses in the earlier part of the decade. However, the most recent update contained no changes in syphilis testing guidelines (*12*), so recent increases are unlikely to result from changes in testing practice. Further, although more MSM were tested for STIs in 2014 compared with 2013, the rate of chlamydia and gonorrhea diagnoses remained relatively stable, while that of syphilis increased.

Given the comprehensive coverage of national STI surveillance and that SHCs in England are open access and free, most syphilis and gonorrhea cases are probably captured in GUMCADv2 (*13*). A key limitation of GUMCADv2 is that it does not collect any behavioral data; however, an enhancement to collect data on behavior, including recreational drug use and unprotected anal intercourse, is being piloted (https://www.gov.uk/guidance/genitourinary-medicine-clinic-activity-dataset-gumcadv3-pilot).

A focus on biomedical interventions, such as preexposure prophylaxis, for the control of HIV among MSM may have unintended consequences for transmission of other STIs, which highlights a need to ensure that robust STI prevention and control measures are in place (*14*). These measures should include promoting condom use; increased screening (in the United Kingdom, quarterly HIV/STI testing of MSM who engage in condomless sex with new partners is recommended); ensuring that services are easily accessible; and promoting other risk reduction strategies to improve the health and well-being of MSM (*15*).
